# Synthesis and highly visible-induced photocatalytic activity of CNT-CdSe composite for methylene blue solution

**DOI:** 10.1186/1556-276X-6-398

**Published:** 2011-05-26

**Authors:** Ming-Liang Chen, Won-Chun Oh

**Affiliations:** 1 Department of Advanced Materials Science & Engineering, Hanseo University, Seosan-si, Chungnam-do, 356-706, Korea

## Abstract

Carbon nanotube-cadmium selenide (CNT-CdSe) composite was synthesized by a facile hydrothermal method derived from multi-walled carbon nanotubes as a stating material. The as-prepared products were characterized by X-ray diffraction, scanning electron microscopy with energy dispersive X-ray analysis, transmission electron microscopy (TEM), and UV-vis diffuse reflectance spectrophotometer. The as-synthesized CNT-CdSe composite efficiently catalyzed the photodegradation of methylene blue in aqueous solutions under visible-light irradiation, exhibiting higher photocatalytic activity.

## Introduction

Environmental problems such as toxic organic pollutants provide the impetus for fundamental and applied research into environmental areas. Semiconductor photocatalysts have attracted considerable attention for a long time in the fields of photochemistry [1-5] because of their usefulness with regard to solving environmental problems. Over the last few years, considerable efforts have been made in the controlled synthesis of various nanoscaled materials to improve their properties for photocatalysis. Cadmium selenide (CdSe) is an n-type semiconductor. Its bandgap energy was reported to be in the range from 1.65 to 1.8 eV [6-9]. CdSe was found to be suitable for various optoelectronic applications such as light-emitting diodes, laser diodes [10-13], catalysis [14], solar cells [15], and biological labeling [16].

More recently, many groups have synthesized CdSe nanomaterials with high photocatalytic activity in the degradation of organic pollutants under UV light irradiation, such as CdSe-Pt nanorods and nanonets [16], hybrid CdSe-Au nanodumbbells [17], CdSe/ZnS-photosensitized nano-TiO_2 _film [18]. Therefore, as an important semiconductor, CdSe is an effective catalyst for photocatalytic degradation of organic pollutants. However, a few recent papers have discussed the preparation and properties of CdSe combining with carbon nanotubes (CNTs) composite. Since the discovery of the CNTs [19,20], they have attracted much attention because their unique mechanical, optical, and electrical properties that may impact many fields of science and technology [21-24]. However, the functionalization of CNTs requires chemical modification of their surface, in order to form the functional groups on the surface.

In this paper, the multi-walled carbon nanotubes (MWCNTs) were used as start material and functionalized by *m*-chlorperbenzoic acid (MCPBA). Then the CNT-CdSe composite were prepared directly via a conventional hydrothermal method. The intrinsic characteristics of resulting composite were studied by X-ray diffraction (XRD), scanning electron microscopy (SEM) with energy dispersive X-ray (EDX), transmission electron microscopy (TEM) analysis and UV-vis diffuse reflectance spectrophotometer. The photocatalytic activity of the as-synthesized samples was evaluated by degrading methylene blue (MB) under irradiation of visible light.

## Experimental

### Materials

Crystalline MWCNTs powder (diameter, 5~20 nm; length, ~10 μm) of 95.9 wt.% purity from Carbon Nano-material Technology Co., Ltd., Pohang-si, Gyungbuk-do, Korea was used as a starting material. For the oxidization of MWCNTs, MCPBA was chosen as the oxidizing agent which purchased from Acros Organics, New Jersey, USA. Benzene (99.5%) was used as the organic solvent which purchased from Samchun Pure Chemical Co., Ltd, Seoul, Korea. Cadmium acetate dihydrate (Cd(CH_3_COO)_2_, 98%), selenium metal powder, and ammonium hydroxide (NH_4_OH, 28%) were purchased from Dae Jung Chemicals & Metal Co., Ltd, Siheung-si, Gyonggi-do, Korea. Anhydrous purified sodium sulfite (Na_2_SO_3_, 95%) was purchased from Duksan Pharmaceutical Co., Ltd, Ansan-si, Gyeonggi-do, Korea. The MB (C_16_H_18_N_3_S·Cl, 99.99+%) was used as model pollutant which purchased from Duksan Pure Chemical Co., Ltd, Ansan-si, Gyeonggi-do, Korea. All chemicals used without further purification and all experiments were carried out using distilled water.

### Synthesis of CdSe and CNT-CdSe composite

#### Synthesis of CdSe

For the synthesis of CdSe compound, the sodium seleno sulfite (Na_2_SeSO_3_) solution and Cd(NH_3_)_4_^2+ ^solution was prepared at first. Na_2_SO_3 _(5 g) and selenium metal powder (0.5 g) were dissolved in 30-mL distilled water and refluxed for 1 h to form Na_2_SeSO_3 _solution. Meanwhile, Cd(CH_3_COO)_2 _(0.5 g) was dissolved in 2-mL distilled water. NH_4_OH (6 mL) was added to it and the mixture was stirred till it dissolved completely to form Cd(NH_3_)_4_^2+ ^solution. Finally, Cd(NH_3_)_4_^2+ ^and Na_2_SeSO_3 _solutions were mixed together and the mixture was stirred and refluxed for at least 5 h. After the temperature of the mixture was brought down to room temperature, the mixture was filtered through Whatman filter paper. The solid obtained was collected and washed with distilled water for five times. After being dried in vacuum at 353 K for 8 h, the CdSe compound was obtained.

#### Synthesis of CNT-CdSe composite

For preparation of the CNT-CdSe composite, the MWCNTs had to functionalize by MCPBA at first. MCPBA (1 g) was melted in 60 mL benzene, and then 0.5 g MWCNTs was put into the oxidizing agent. The mixture was stirred with a magnet for 6 h at 343 K. Then the MWCNTs was dried at 373 K and spared.

**Figure 1:**
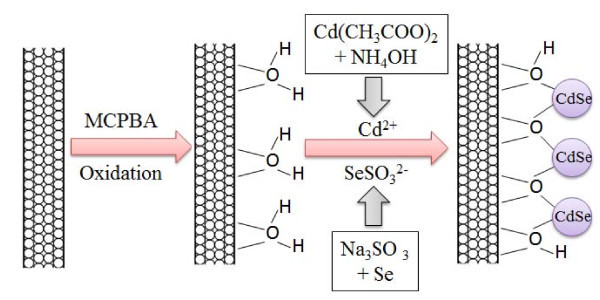
**Schematic presentation of the functionalization of MWCNTs and the coupling of CdSe nanoparticles with MWCNTs**.

The functionalized MWCNTs with Cd(NH_3_)_4_^2+ ^and Na_2_SeSO_3 _solutions which were prepared above were mixed together and the mixture was stirred and refluxed for at least 5 h. After the temperature of the mixture was brought down to room temperature, the mixture was filtered through Whatman filter paper. The solid obtained was collected and washed with distilled water for five times. After being dried in vacuum at 353 K for 8 h, the CNT-CdSe composite with chemical band was obtained. Figure 1 shows the schematic presentation of the functionalization of MWCNTs and the coupling of CdSe nanoparticles with MWCNTs.

### Characterization

XRD (Shimadz XD-D1, Uki, Kumamoto, Japan) result was used to identify the crystallinity with monochromatic high-intensity CuKα radiation (*λ *= 1.5406 Å). SEM (JSM-5600, JEOL Ltd., Tokyo, Japan) was used to observe the surface state and structure of prepared composite using an electron microscope. Transmission electron microscopy (TEM, Jeol, JEM- 2010, Japan) was used to determine the state and particle size of prepared composite. TEM at an acceleration voltage of 200 kV was used to investigate the number and the stacking state of graphene layers on various samples. TEM specimens were prepared by placing a few drops of sample solution on a carbon grid. The element mapping over the desired region of prepared composite was detected by an EDX analysis attached to SEM. UV-vis diffuse reflectance spectra were obtained using an UV-vis spectrophotometer (Neosys-2000, Scinco Co. Ltd., Seoul, Korea) by using BaSO_4 _as a reference at room temperature and were converted from reflection to absorbance by the Kubelka-Munk method.

### Photocatalytic activity measurements

The photocatalytic activity under visible lamp (KLD-08L, 220 V, 50-60 Hz, 8 W, pure white, *λ *> 420 nm, Fawoo Tech Co., Ltd., Tokyo, Japan) irradiation of the CNT-CdSe composite was evaluated by using MB as the model substrate. In an ordinary photocatalytic test performed at room temperature, 0.05 g CNT-CdSe composite was added to 50 mL of 1.0 × 10^-5^-mol/L MB solution, which was hereafter considered as the initial concentration (*c*_0_). Before turning on the visible lamp, the solution mixed with composite was kept in the dark for at least 2 h, allowing the adsorption/desorption equilibrium to be reached. Then, the solution was irradiated with visible lamp. The first sample was taken out at the end of the dark adsorption period (just before the light was turned on), in order to determine the MB concentration in solution after dark adsorption, which was hereafter considered as the initial concentration (*c*_ads_). Samples were then withdrawn regularly from the reactor by an order of 30, 60, 90, 120, 180, and 240 min, and immediately centrifuged to separate any suspended solid. The clean transparent solution was analyzed by using a UV-vis spectrophotometer (Optizen POP, Mecasys Co., Ltd, Seoul, South Korea) at wavelength of 665 nm [25-27].

## Results and discussion

### Characterization

**Figure 2:**
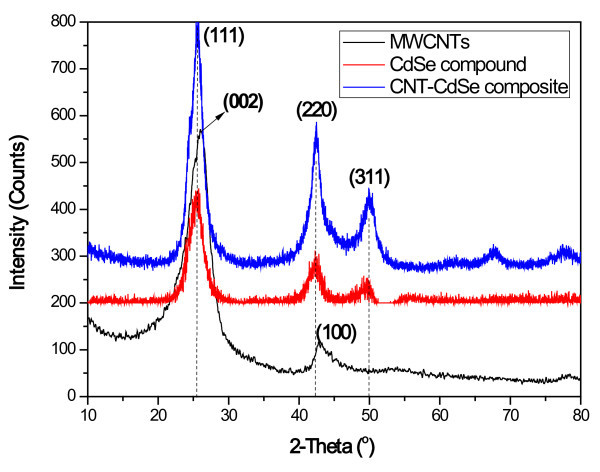
**XRD patterns of MWCNTs, CdSe, and CNT-CdSe composite**.

Figure 2 shows X-ray patterns of the pristine MWNTs, CdSe, and CNT-CdSe composite. From Figure 2, it can be seen that the diffractogram of pure MWCNTs exhibit the typical peaks at 25.9° and 42.7°, corresponding to the graphite (002) and (100) reflections (Joint Committee for Powder Diffraction Studies (JCPDS) No. 01-0646) [28], respectively. For CdSe compound, the XRD diffraction peaks around 2*θ *of 25.4°, 42°, and 50°, which can be indexed to the characteristic peaks (111), (220), and (311) plane reflections of cubic crystal structure CdSe with lattice constants of 6.05 Å according to the standard powder diffraction data (JCPDS No. 65-2891 for CdSe, cubic) [29,30]. However, for CNT-CdSe composite, only the typical peaks arose from CdSe were detected. As we known, CdSe has three crystalline forms wurtzite (hexagonal), sphalerite (cubic), and rock-salt (cubic). The sphalerite CdSe structure is unstable and converts to the wurtzite form upon moderate heating. The transition starts at about 130°C, and at 700°C it completes within a day. The rock-salt structure is only observed under high pressure. However, in our study, the highest temperature was 70°C to approximately 80°C at hydrothermal experiment. So the obtained CdSe compound and CNT-CdSe composite exited cubic CdSe structure. Therefore, the micromorphology of CNT-CdSe is different from that of the mixture of MWNTs and CdSe. No peaks for impurities are detected, indicating that the hydrothermal method used in this study is responsible for the formation of the CNT-CdSe composite.

**Figure 3:**
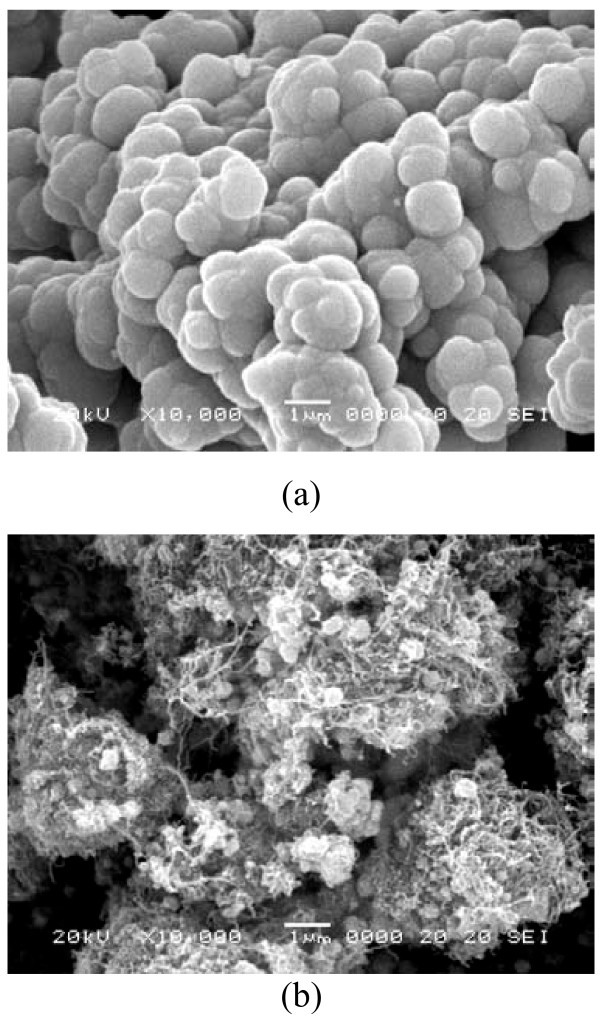
**SEM microphotographs of CdSe (a) and CNT-CdSe (b) composite**.

**Figure 4:**
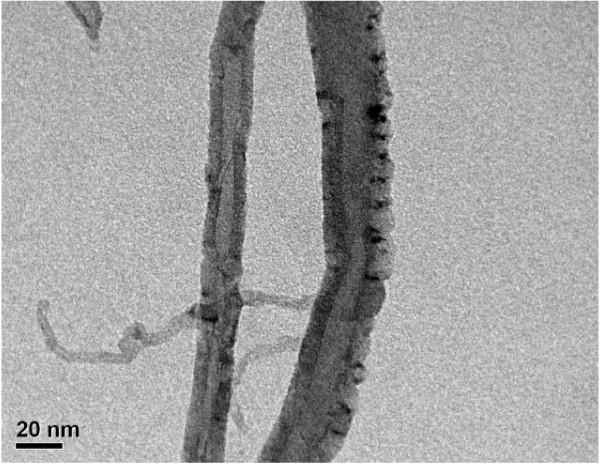
**TEM image of CNT-CdSe composite**.

Figure 3 shows the SEM microphotographs of CdSe and CNT-CdSe composite. From the Figure 3a, very uniform spherical-shaped CdSe particles with agglomerate together can be observed. For CNT-CdSe composite, as shown in Figure 3b, spherical-shaped agglomerated CdSe particles are mixed with MWCNTs. More detailed information of the surface state can be confirmed by the transmission electron microscopy (TEM). Figure 4 shows the TEM image of CNT-CdSe composite. It can be observed that the surface of MWCNTs have been coated with CdSe layers uniformly with particle size of about 10 nm.

**Figure 5:**
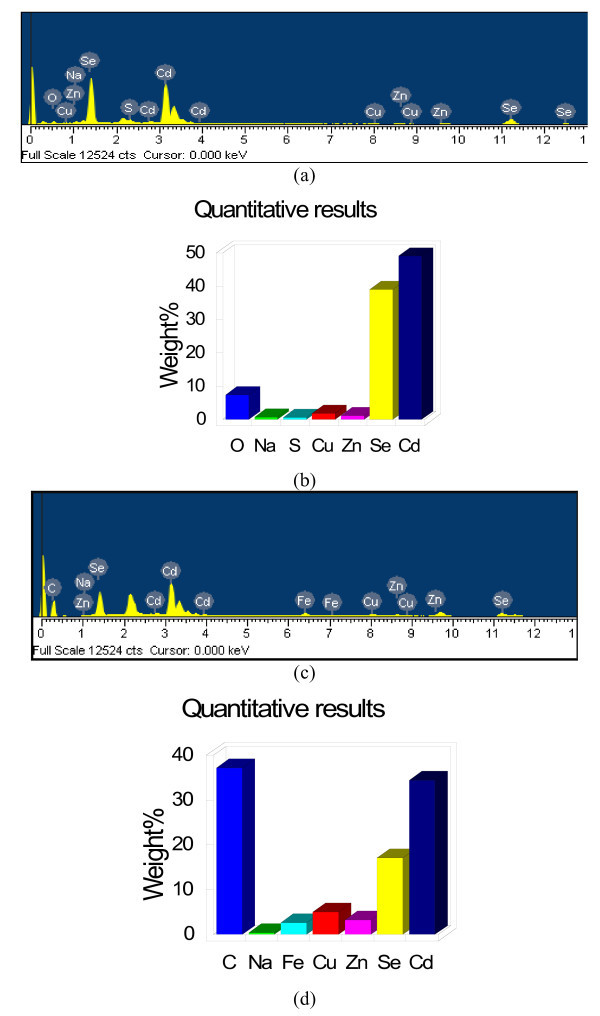
**EDX microanalysis and element weight percentageof CdSe ((a) and (b)) and CNT-CdSe ((c) and (d))**.

To get information about change in elements and element weight percent, the prepared CdSe and CNT-CdSe composite were examined by EDX. Figure 5 shows the EDX microanalysis and element weight percent of CdSe and CNT-CdSe composite. From Figure 5a, b, main elements such as Cd and Se are existed in CdSe composite. Apart from these two kinds of main elements, the main element C is also existed in CNT-CdSe composite, as shown in Figure 5c, d.

**Figure 6:**
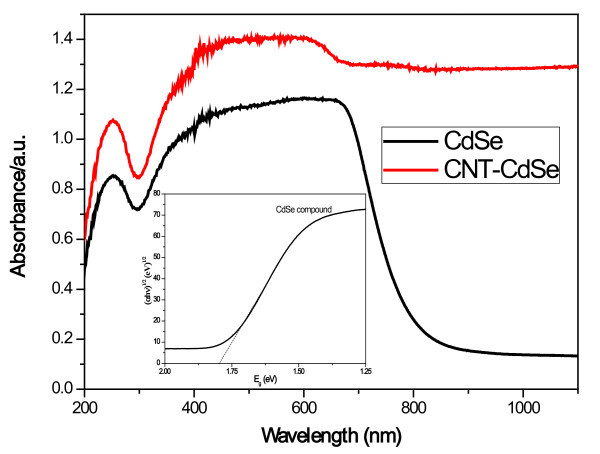
**UV-vis diffuse reflectance spectra of CdSe and CNT-CdSe composite**.

Figure 6 shows the UV-vis diffuse reflectance spectra of CdSe and CNT-CdSe composite. The reflectance characteristics of the CdSe composite were quite similar to that of the CNT-CdSe composite except the CdSe composite has an absorption edge at 830 nm. We can use the following formula to calculate the band gap energy of CdSe.

where *α*, *v*, *E*_*g*_, and *A *are the absorption coefficient, light frequency, band gap, and a constant, respectively. Therefore, the band gap energy (*E*_*g*_) of CdSe compound can be estimated from a plot of (*αhv*)^1/2 ^versus photo energy (*hv*), as shown in figure inset in Figure 6. The band gap energy of CdSe is 1.74 eV, which is fairly close to literature value of 1.65 to 1.8 eV (CdSe) [6-9].

Moreover, the two composite both exhibit strong absorption in the UV light region with wavelength less than 400 nm and visible-light region with wavelength at 400-800 nm, assigned to the band adsorption of CdSe. And the absorption of CNT-CdSe composite is higher than that of CdSe compound in both of UV light and visible-light region, as the MWCNTs act as good electron acceptors can accept the electrons from light irradiation [31,32], indicating the CNT-CdSe composite would exhibit more excellent photoactivity than CdSe compound.

### Degradation of MB solution

The photocatalytic activities of the CNT-CdSe composite were evaluated by the photodegradation of MB aqueous solution under visible-light irradiation. The decreasing concentration of MB in the photocatalytic reaction was used to evaluate the activity of the composite. The characteristic absorption peak of MB solution at 665 nm was chosen as the monitored parameter to detect the concentration of MB solution.

**Figure 7:**
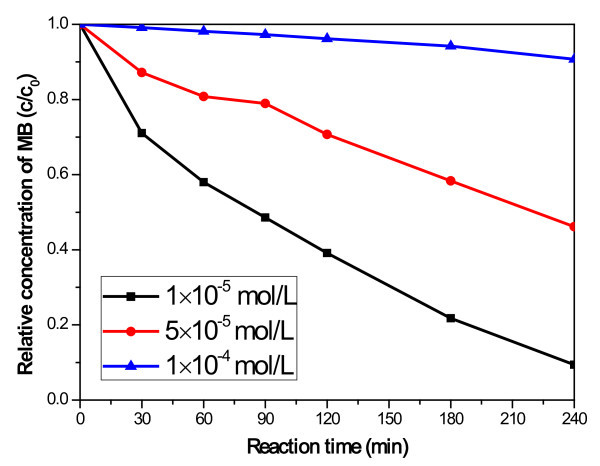
**Dependence of the MB concentration of the CNT-CdSe composite (0.05 g) under visible light irradiation**.

Figure 7 represents the degradation of MB over CNT-CdSe composite with amount of 0.05 g as a function of the original MB concentration under visible-light irradiation. For different concentrations of the original MB aqueous solution, the level of photodegradation is quite different after 240 min illumination. After illumination for 240 min, the adsorption efficiency of the 1 × 10^-5 ^mol/L and 5 × 10^-5 ^mol/L was 91% and 54%, respectively. However, for the 1 × 10^-4 ^mol/L and MB concentration, only about 10% was degraded after 240 min. Therefore, it seems that the photodegradation efficiency of the MB photocatalyzed by the CNT-CdSe composite decreased as the original MB concentration increased. The main reason is that the initial dye concentration may affect strongly the rate of the photocatalytic process.

**Figure 8:**
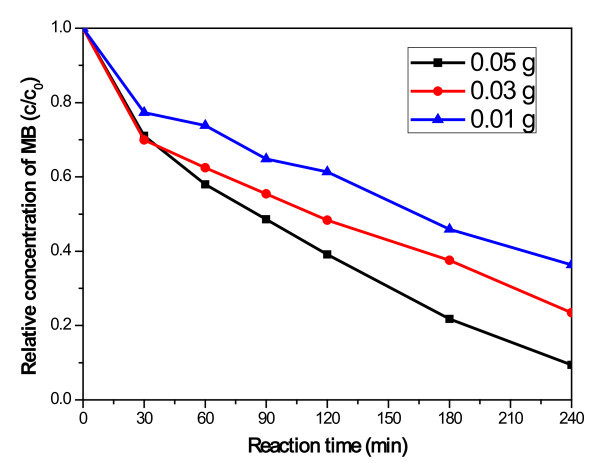
**Degradation of MB under visible light irradiation for the CNT-CdSe composite with different amount**.

Figure 8 shows the effect of the amount of the CNT-CdSe composite on the photocatalytic performance under visible-light irradiation. The concentration of MB solution is 1×10^-5 ^mol/L. From the Figure 8, it is obvious that 0.05 g of the CNT-CdSe composite gave the best results of photodegradation of MB solution. And the photodegradation efficiency of the MB photocatalyzed by the CNT-CdSe composite decreased as the amount of the CNT-CdSe composite increased.

**Figure 9:**
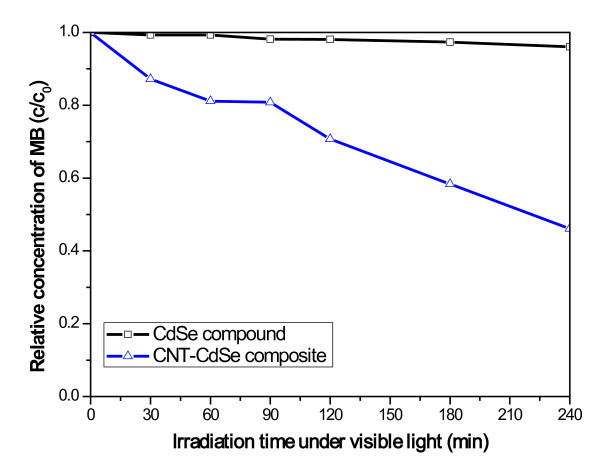
**Degradation of MB under visible light irradiation for the CdSe compound and CNT-CdSe composite**.

Figure 9 represents the degradation of MB over CdSe compound and CNT-CdSe composite under visible-light irradiation, the MB concentration is 1×10^-5 ^mol/L; the amount of CdSe compound and CNT-CdSe composite is 0.05 g. We can clearly see that the concentration of the MB solution gradually diminish with increasing irradiation time for all of samples. After irradiation for 240 min, the CdSe compound has almost no photocatalytic activity toward the photodegradation of MB solution. The presumed reason is that a mass of visible light may be absorbed by the MB molecules in aqueous solution rather than the CdSe particles for high MB concentration, which can reduce the efficiency of the catalytic reaction. However, for CNT-CdSe composite, a much excellent photocatalytic activity toward the photodegradation of MB solution can be observed and the MB concentration is removed 55% after irradiation under visible light for 240 min.

**Figure 10:**
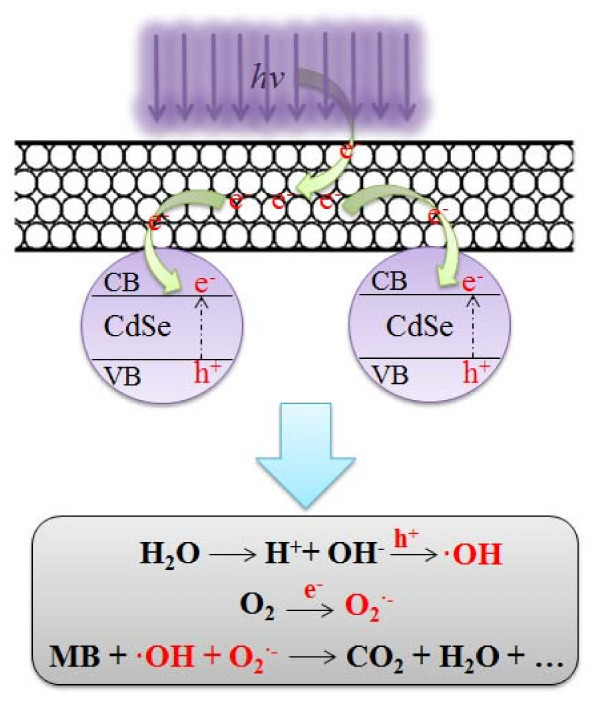
**MWCNTs acting an electron acceptor for improving the photocatalytic activity of CdSe compound**.

According to the above experimental data, the CNT-CdSe composite has an excellent photocatalytic activity toward the photodegradation of MB solution under visible-light irradiation. Figure 10 shows the MWCNTs acting an electron acceptor for improving the photocatalytic activity of CdSe compound. Under irradiation by visible lamp, the MWCNTs acting as good electron acceptors can accept the electrons by light irradiation [31,32]. Meanwhile, the CdSe can be also excited to produce the electrons and holes in the conduction band (CB) and valence band of CdSe. Then the electrons accepted by MWCNTs from light can transfer into the CB of CdSe, thereby increasing the number of electrons as well as the rate of electron-induced redox reactions. The generated electrons (e^-^) probably react with dissolved oxygen molecules and produce oxygen peroxide radical O_2_^•-^, the positive charged hole (h^+^) may react with the OH^- ^derived from H_2_O to form hydroxyl radical OH•. The MB molecule then can be photocatalytically degraded by oxygen peroxide radical O_2_^•- ^and hydroxyl radical OH• to CO_2_, H_2_O, and other mineralization [31-34].

## Conclusions

In this study, CNT-CdSe composite was successfully synthesized by a simple hydrothermal method. From the XRD patterns, the cubic crystal structure of CdSe can be observed. TEM image shows that the surface of MWCNTs has been coated with CdSe layers uniformly with particle size of about 10 nm. The EDX results reveal the presence of C, Cd, and Se with high content in prepared composite. The diffuse reflectance spectra suggest the CNT-CdSe composite shows strong photoabsorption at UV light and visible-light range. The photocatalytic activity of the CNT-CdSe composite is investigated by degradation of MB in aqueous solution under visible-light irradiation. The results reveal that CNT-CdSe composite exhibit excellent photocatalytic activity for degradation of MB solution under visible-light irradiation.

## Competing interests

The authors declare that they have no competing interests.

## Authors' contributions

WCO conceived of the study, and participated in its design and coordination. MLC carried out the experiment, processed the data, wrote and submitted the manuscript. All authors read and approved the final manuscript.
